# Strategies to prevent anastomotic leakage after esophagectomy and gastric conduit reconstruction

**DOI:** 10.1007/s00423-020-01926-8

**Published:** 2020-07-10

**Authors:** Diana Vetter, Christian A. Gutschow

**Affiliations:** Division Head Upper Gastrointestinal Surgery, Department of Visceral and Transplant Surgery, University Hospital Zurich, Rämistrasse 100, 8091 Zurich, Switzerland

**Keywords:** Esophagectomy, Anastomotic leakage, Prevention

## Abstract

**Background:**

Surgery remains the cornerstone of esophageal cancer treatment but is burdened with high procedure-related morbidity. Anastomotic leakage as the most important surgical complication after esophagectomy is a key indicator for quality in surgical outcome research.

**Purpose:**

The aim of this narrative review is to assess and summarize the current knowledge on prevention of anastomotic leakage after esophagectomy and to provide orientation for the reader in this challenging field of surgery.

**Conclusions:**

There are various strategies to reduce postoperative morbidity and to prevent anastomotic leakage after esophagectomy, including adequate patient selection and preparation, and many technical-surgical and anesthesiological details. The scientific evidence regarding those strategies is highly heterogeneous, ranging from expert’s recommendations to randomized controlled trials. This review is intended to serve as an empirical guideline to improve the clinical management of patients undergoing esophagectomy with a special focus on anastomotic leakage prevention.

## Introduction

Despite significant progress in perioperative management, esophagectomy for cancer remains a procedure with relevant morbidity, even in high-volume centers [1, 2]. The spectrum of postoperative morbidity after esophagectomy is broad, with pulmonary and anastomotic complications being the most common types [3–5].

Although different definitions and classifications [6] have been described, anastomotic leakage (AL) is a key indicator for surgical quality [1, 7] owing to its correlation with surgical expertise [8] and its tremendous impact on postoperative outcome. Sound surgical technique is key for proper anastomotic healing; however, AL rates > 20% have been reported even by renowned centers of expertise [9, 10]. Moreover, there is no evidence that new technology such as minimally invasive and robotic-assisted techniques would reduce the risk for AL.

Various strategies aim at prevention of AL, including proper patient selection, preparation and prehabilitation programs, techniques to improve and control vascularization of the gastric conduit, and many surgical and anesthesiological details. In this context, the aim of this narrative review was to summarize the spectrum of these strategies and to comment on their scientific evidence and clinical significance.

## Patient selection and preparation

### Neoadjuvant treatments, comorbidity, and age

Neoadjuvant chemotherapy or chemo-radiation followed by surgery has become the standard of care in the treatment of esophageal cancer [11, 12]. However, a recent large retrospective European multicenter study revealed a significantly higher risk of postoperative complications including AL in cardiorespiratory comorbid patients after neoadjuvant chemo-radiation, but not after neoadjuvant chemotherapy [13, 14]. Consequently, neoadjuvant chemo-radiation should be employed with particular caution in patients with known respiratory comorbidity [15].

Numerous comorbidities are linked to increased AL risk such as obesity, heart failure, coronary artery disease, peripheral vascular disease, hypertension, steroids, diabetes mellitus, renal insufficiency, and tobacco use [16]. In addition, atrial fibrillation [17] and COPD [18] are known independent risk factors. Most comorbidities have a negative impact on microvascular perfusion, and it has been hypothesized that arteriosclerosis may play an important role in the etiology of AL [19]. Consequently, several retrospective cohort studies have confirmed an association between AL and loco-regional post-coeliac [20, 21] and aortic and coeliac trunk [22] calcifications. Moreover, others have evidenced an association between AL and supra-aortic and coronary arteriosclerosis [18], implying that general radiological arteriosclerosis scores may be useful to estimate the risk of AL [21, 23, 24].

Besides the abovementioned factors, age may play a major role in postoperative morbidity as older patients have more comorbidities and a reduced physiological resilience [25]. For example, older individuals have a higher probability of new-onset atrial fibrillation after esophagectomy [26], which is a known risk factor for pneumonia and AL. In addition, in patients ≥ 75 years, the nutritional status is often impaired [27] and the sarcopenia rate is higher [28, 29]. However, none of the prospective cohort studies comparing younger (< 75 years) and older (≥ 75) cohorts found an association between age and postoperative morbidity or mortality [27, 30].

In summary, several secondary illnesses and comorbidities play a pivotal role in patient outcome after esophagectomy. In order to optimize preoperative assessment of esophageal cancer patients, prediction tools using readily available characteristics have been suggested recently [31]. Careful patient selection and thorough navigation through risks and benefits remains key for achieving optimal results.

### Preoperative nutrition

Malnutrition is highly prevalent in patients with esophageal carcinoma and has been linked to a higher incidence of AL in GI-tract surgery [32]. Consequently, nutritional support prior to surgery is associated with a reduced complication rate after esophagectomy [33]. In this context, screening of the nutritional status in patients scheduled for esophagectomy following the ESPEN (European Society for Clinical Nutrition and Metabolism) criteria is highly recommended [34]. Most experts agree that preoperative nutritional support is indicated if body weight loss was ≥ 10–15% over the past 6 months, and in patients with a BMI < 18.5 kg/m^2^ or a serum albumin < 30 g/l [34]. In addition, nutritionists should be involved to monitor protein and calorie intake and to assess the need for dietary supplements. In case of severe dysphagia, placement of an enteral feeding tube and preoperative nutritional support for a minimum of 7–14 days is recommended according to the ESPEN guidelines [35, 36].

Perioperative dietary supplementation with immune-stimulating nutrients (omega-3 fatty acids, arginine, nucleotides) may reduce oxidative stress and inflammatory response. Correspondingly, perioperative immunonutrition was found to reduce postoperative morbidity in gastrointestinal cancer surgery in a systematic review of randomized controlled trials (RCTs) [37]. However, another recent meta-analysis of RCTs with a specific focus on esophagectomy did not confirm a positive effect of immunonutrition on AL, overall morbidity, postoperative hospital stay, or immune indices such as C-reactive protein, interleukin-6, IL-8, and tumor necrosis factor-α [38]. Therefore, immunonutrition prior to esophageal cancer surgery remains a controversial issue.

### Prehabilitation

Prehabilitation is a relatively new concept that entails a variety of preoperative measures to prepare patients for surgery. Prehabilitation is part of enhanced recovery after surgery (ERAS) protocols [39] and includes nutritional, physical, and psychological components. The physical component of prehabilitation programs entails inspiratory muscle training, aerobic exercise, and general strengthening activities. The effect of preoperative inspiratory muscle training on postoperative morbidity and AL rate remains controversial, although most research points towards a beneficial effect. The use of spirometers has been found to correlate positively with postoperative pulmonary morbidity [40], and two RCTs and one observational study have evidenced a reduction in severe pulmonary [41, 42] and overall complications [43] through perioperative inspiratory muscle training. However, other randomized studies could not confirm a benefit of inspiratory muscle training [44].

Likewise, aerobic exercises and strengthening activities during > 4 weeks prior to esophagogastric surgery had a positive impact on preoperative fitness tests, an effect that sustained for 4–8 weeks after surgery [45]. Four RCTs on prehabilitation programs in gastroesophageal carcinoma are pending [46–49]. Interestingly, an RCT comparing the effect of prehabilitation with postoperative rehabilitation after colorectal cancer surgery evidenced that a 4-week prehabilitation program was even more effective than postoperative rehabilitation [50].

## Anesthesiological management and perioperative monitoring

Anesthesiological measures focus on lung-protective ventilation, fluid management, and analgesia [51]. Restrictive perioperative fluid management has been recommended to reduce the rate of pulmonary complications and AL [51, 52]. However, excessive fluid restriction may cause hypovolemia, hypotension, and need for catecholamines, thus increasing the risk for ischemia and AL [6]. Similarly, epidural anesthesia has become an integral part of ERAS programs [53] and represents the standard of care for perioperative pain control in many centers. However, epidural analgesia causes perioperative hypotension in 20–76% of patients and has been directly associated with AL [54]. Therefore, standardized continuous monitoring of perioperative hemodynamics is pivotal to counterbalance potential drawbacks of esophagectomy-specific anesthesiological management [52].

## Surgical-technical aspects

### Intrathoracic versus cervical anastomosis

There is a long-standing debate whether the anastomosis after gastric pull-up procedure should be placed in the neck or intrathoracically. Most RCTs [55–58], retrospective cohort studies [2, 59, 60], and metaanalyses [21] show higher leak and recurrent nerve palsy rates but a reduced leak-associated morbidity after cervical anastomosis [4, 55, 60, 61]. The higher leak rate in cervical anastomoses might be caused by greater tension and placement in the gastric fundus (with potentially impaired vascular supply), whereas intrathoracic esophagogastrostomies are constructed under less longitudinal stress in a more distal and better-perfused gastric area [7, 62]. On the other hand, management of cervical AL is more straightforward and potentially less life threatening than intrathoracic AL. Interestingly however, a recent international survey among specialized surgeons has evidenced a strong tendency towards intrathoracic reconstructions [63], presumably owing to the substantial improvements regarding anastomotic leak management over the last years [64].

### Gastric tube formation

Intestinal continuity after esophageal resection is typically restored using the whole stomach or a gastric tube. However, there is still considerable debate about the ideal type of conduit. The rationale for using a wide gastric tube or even the whole stomach was established more than 50 years ago by Levasseur and Couinaud [65] who showed that the intramural gastric vascular network originating from the left gastric artery connects the right and left gastroepiploic perfusion areas between antrum and fundus. This finding was later confirmed in autopsy studies [66, 67]. Accordingly, removal of the lesser curvature during gastric tubulization may induce ischemia of the upper part of the stomach and increase the risk for AL. With this in mind, others [68] have suggested to only remove the upper part of the greater curvature and to place the staple-line close to the lesser curvature in order to preserve as much intramural vascular network as possible. Jean-Marie Collard, from the same point of view, found that vascular and lymphatic denudation of the lesser curvature provides a similar lengthening effect without destruction of the intramural vascular network and advocated the whole stomach as best option for esophagogastric reconstruction with lower AL rate and superior function [67]. However, this concept has been challenged, arguing that a long and narrow gastric tube could reduce the incidence of AL owing to lower anastomotic tension. In summary, there is conflicting data in the literature regarding AL rate after narrow and wide gastric tube or whole stomach reconstructions. A recent meta-analysis found a similar AL rate, but a higher incidence of gastroesophageal reflux and thoracic stomach syndrome after whole stomach reconstruction [69]. Accordingly, most experts currently use a relatively broad (4–5 cm) gastric tube [70].

### Anastomotic technique

Many different techniques have been devised for reconstruction after esophagectomy: (semi)mechanical or hand-sewn, linear-stapled, circular-stapled and double-stapled, end-to-side, side-to-side, single-row, and double-row techniques. Moreover, the number of individual variations is legion owing to different suture materials and stapling devices, and the manifold individual technical steps taken by each surgeon. In addition, with the advent of minimally invasive esophagectomy, adjustments and modifications of existing techniques and even completely new designs have been developed [63].

A recent metaanalysis [71] showed similar AL rates in end-to-side circular-stapled and side-to-side linear-stapled esophagogastrostomy. In contrast, AL rates after side-to-side linear-stapled esophagogastrostomy were lower compared with the end-to-side hand-sewn technique for cervical, but not for intrathoracic, anastomoses [72]. Consistently, both studies revealed a lower rate of anastomotic stenosis of side-to-side linear-stapled techniques, but the significance of these results remains doubtful owing to a lack of uniform definitions of AL and stricture.

In a large retrospective multicenter study including 966 patients that underwent minimally invasive thoracoscopic-laparoscopic esophagectomy, leakage rates after intrathoracic end-to-side double-stapling (23.3%) and cervical end-to-side hand-sewn (25.1%) techniques were significantly higher compared with intrathoracic side-to-side linear (15.6%), end-to-side purse-string (13.9%), and cervical side-to-side linear-stapled esophagogastrostomy (11.8%) [7]. Multivariable analysis confirmed anastomotic technique as independent predictor of leakage. However, the authors concluded that the above results require cautious interpretation considering the long learning curve of minimally invasive esophagectomy. In conclusion, the scientific evidence on this topic remains weak and superiority of certain techniques in terms of leakage rate is questionable. Therefore, a surgeon’s choice of a specific anastomotic design remains mainly based on personal experience rather than on solid scientific proof.

### Additional surgical measures

Pedicled omental flaps have proven their usefulness in the management of complex thoracic and abdominal fistula. After esophagectomy, the upper part of the omentum along the gastric fundus can easily be wrapped around and sutured to the anastomosis [73]. This technical maneuver may be used in both cervical and intrathoracic anastomosis [74]. The well-perfused fatty tissue provides ample supply of nutrition and oxygen to the anastomotic area including secretion of vascular endothelial growth factor for enhanced angiogenesis and oxygenation [75]. In case of leakage, the omental flap may cover the defect by creating a contained situation that prevents free leakage to the mediastinum.

Numerous studies including a recent meta-analysis of randomized trials [76] have evaluated omental wrapping with positive results, and most specialist surgeons endorse this adjunct to improve healing of esophagogastric reconstructions.

With a similar rationale, many surgeons recommend to suture the mobilized pleura over the completed anastomosis [77]. “Pleural tenting” may be applied in combination with an omental wrap or as a single additional maneuver. However, to our best knowledge, this procedure has not been evaluated in comparative studies and remains a topic in need of further investigation.

Excessive tension at the anastomosis may occur in case of an incomplete vascular arcade, previous gastric surgery, or in very high cervical anastomoses. In these situations, additional length of the gastric tube can be gained by division of the peritoneal reflection of the hepatoduodenal ligament and by extensive duodenal mobilization (Kocher’s maneuver). These techniques are particularly helpful if the distance between pylorus and hiatus remains too large after gastric mobilization [78]. In extreme cases, division of the branches of the right gastric artery, or even duodenal [79] or pyloric [80] diversion on a jejunal limb may generate extra mobility. Similarly, longitudinal [81] or circular [82] incision of the gastric serosa has been described to achieve additional length. However, we are not aware of any research on this topic and indications remain entirely based on individual experience.

### Intraoperative perfusion monitoring

Assessment of the viability of the neo-esophagus usually relies on the surgeon’s subjective evaluation. Typical signs of a healthy gastric tube include rosy-colored tissues, a pulsating vascular arcade, and active bleeding from the staple line or the upper part of the omentum. However, local ischemia at the tip of the gastric interponate is a frequent finding after gastric mobilization. Ischemia may depend on vascularization and other factors such as vasoconstriction, fluid management, catecholamine dosage, and the patient’s hemodynamic situation. Therefore, a range of innovative tools for assessment of gastric tube viability has been developed over the last years: Laser Doppler flowmetry, near infrared spectroscopy, optical coherence tomography, and laser speckle contrast, infrared thermographic, fluorescence, and hyperspectral imaging [83]. Preliminary results of retro- and prospective cohort studies have shown promising results [84], and some of these tools have become commercially available. Perfusion monitoring is a promising technique, and a literature review has evidenced a significant benefit regarding AL rate [85]. Various questions remain albeit unanswered because many technical solutions still rely on subjective assessment, and no general agreement on perfusion parameters, normal values, thresholds, or quantitative validation has been established. Therefore, the current evidence supporting a routine intraoperative use of perfusion monitoring is still pending, and further research is required to fully explore its clinical potential.

### Ischemic conditioning

Partial gastric devascularization prior to esophagectomy is a relatively new concept in esophageal surgery. The idea of ischemic conditioning is to optimize the gastric blood flow in preparation for later esophagectomy through preoperative selective occlusion of the left gastric ± short gastric arteries. Arterial occlusion is accomplished by either surgical ligation or interventional embolization [86]. The resulting relative ischemia is expected to resolve via hemodynamic redistribution from the remaining vessels, thus avoiding ischemia during later reconstruction. In addition, gastric devascularization may lead to a demarcation of ischemic areas, facilitating the choice of the best location for anastomotic reconstruction [87]. Shortcomings of gastric conditioning include the need for additional resources, increased cost [88], and adhesion formation complicating subsequent gastric mobilization and lymphadenectomy [89, 90]. Ischemic conditioning has been investigated in prospective and retrospective cohort studies including several RCTs [86]. Owing to divergent technical approaches, it is very difficult to draw conclusions. However, two recent meta-analyses of the current literature did not reveal a significant impact on AL rates [86, 88]. Therefore, many early advocates have abandoned ischemic conditioning in routine cases and reserve this option for high-risk patients that might benefit from a two-stage approach [91].

### Gastric decompression

According to Laplace’s law, the wall tension in a cylindrical vessel increases with greater radius and higher luminal pressure (wall tension = pressure × radius). By applying basic physics to surgical practice, the intramural strain of a dilated gastric tube will be higher than in a properly drained interponate. Accordingly, insufficient postoperative gastric decompression may cause impaired blood perfusion, reduced oxygen supply, and a higher risk for AL or even gastric tube necrosis. Another potential benefit of gastric decompression is the prevention of regurgitation and aspiration. Therefore, most specialists rely on routine postoperative drainage of the conduit. On the other hand, prolonged gastric drainage may interfere with ERAS programs through delayed oral feeding with longer hospital stay and increased cost. In addition, nasogastric drains may cause discomfort and can also induce aspiration [92]. Therefore, some surgeons prefer percutaneous transcervical [93] or retrograde [94, 95] (via drain-jejunostomy) conduit decompression. To untangle potential associations between postoperative conduit drainage and complications, numerous studies including several RCTs have been conducted with divergent results. A recent review on preoperative, early or late postoperative removal of gastric drains revealed no effect on AL rate, pulmonary complications, or mortality [96]. However, the current evidence remains weak owing to small sample sizes and the heterogeneity of the available evidence. In addition, comparability is further limited due to different techniques of luminal decompression, including single- or double-lumen (sump-)tubes with or without application of varying negative pressure.

### Preemptive vacuum therapy

Treatment of AL has considerably progressed in recent years, and surgical revision has been largely replaced by interventional procedures. In addition to stent placement, endoluminal vacuum therapy (EVT) has become the treatment of choice in many specialized centers [64]. In EVT, a polyurethane sponge connected to a hose is brought to the anastomotic area via endoscopy. After vacuum application, the sponge drains the leakage cavity and removes secretions and necrosis, accomplishing an 80–90% healing rate [97].

A novel idea is to use the EVT technology in a preemptive setting, with the aim of preventing AL and reducing postoperative morbidity. In a recent case series, early EVT in patients with anastomotic ischemia was effective in six of eight esophagectomy patients [98]. Likewise, in a porcine model, intraoperative application of EVT in esophagogastric anastomoses with intentional 1 cm defects resulted in complete healing [99]. Considering the high incidence of AL, we have recently implemented preemptive EVT (pEVT) in patients undergoing esophagectomy and gastric tube reconstruction. Initial results were promising with a low leakage rate of 5% [100]. Since then, we have used pEVT with a persistently low AL rate in more than 80 cases. In the rare event of AL, clinical courses were generally mild and without septic complications. Based on this positive experience, we have designed an international multicenter RCT (NCT04162860). The aim of this project is to prove whether pEVT may reduce AL rates and overall morbidity after minimally invasive esophagectomy.

## Comment

Anastomotic leakage after esophagectomy is a severe complication with potentially devastating consequences. A wide range of measures to prevent AL has been suggested encompassing everything from adequate patient selection and prehabilitation over surgical training to many details of the operative procedure and perioperative management. Unfortunately, the quality of clinical research is highly variable and only few interventions are supported by strong evidence. Therefore, even in high-volume centers, both prevention and management of AL are mostly guided by empiric observation, pragmatism, and personal experience rather than based on solid scientific evidence.

The current lack of standardized definitions for key criteria of surgical quality outcome remains a major issue. As an example, there are more than 50 different definitions for anastomotic leakage [101–103] leaving much room for interpretation and impeding objective surgical outcome research. In this context, the definitions and classifications of complications after esophagectomy elaborated by the ECCG are a major step in the right direction [104] but may still take time to gain wide acceptance.

Since various comorbidities are clearly linked to increased AL incidence, selection of appropriate candidates for surgery might be the most efficient way to reduce postoperative morbidity. However, in many departments, functional evaluation of patients scheduled for esophagectomy is still arbitrary without clear guidelines. Therefore, both straightforward and accurate predictive tools are urgently needed. In this context, the introduction of simple nomograms using readily available clinical parameters to predict complication risks may gain acceptance in the future [31].

Surgical expertise, operative skills, and the use of state-of-the-art technique are the basic ingredients required for a successful esophageal cancer surgery program. Of note, minimally invasive esophagectomy has an extremely long learning curve of more than 100 cases until acceptable postoperative morbidity may be attained [8]. The effect of surgical learning on outcome has been shown based on operative time, overall morbidity and AL rate, percentage of textbook outcomes, and the number of resected lymph nodes [105–111]. Therefore, centralization of esophageal surgery in order to increase case numbers per center is pivotal to reduce the morbidity of this demanding procedure. In addition, learning curricula, proctoring programs, fellowships, and specialized courses with specific training in esophagectomy should be strongly encouraged.

## Data Availability

Not applicable.
